# 

**Published:** 1871

**Authors:** 


☞ *THE JOURNAL will not be sent, after the present number, to any who have not renewed their subscriptions.*

**☞ NOTICE.—During the great fire our subscription book was burned. Subscribers will please send us their names at earliest moment.**

**☞ Remit $3 for the next volume, commencing January, 1872.**

